# Who Was Shopping More During the Spring Lockdown 2020 in Germany?

**DOI:** 10.3389/fpsyt.2021.650989

**Published:** 2021-03-30

**Authors:** Ekaterini Georgiadou, Anne Koopmann, Astrid Müller, Tagrid Leménager, Thomas Hillemacher, Falk Kiefer

**Affiliations:** ^1^Department of Psychiatry and Psychotherapy, Paracelsus Medical University, Nuremberg, Germany; ^2^Department of Addictive Behavior and Addiction Medicine, Central Institute of Mental Health, Medical Faculty Mannheim/Heidelberg University, Mannheim, Germany; ^3^Feuerlein Center on Translational Addiction Medicine (FCTS), University of Heidelberg, Heidelberg, Germany; ^4^Department of Psychosomatic Medicine and Psychotherapy, Hannover Medical School, Hannover, Germany; ^5^Department of Psychiatry, Social Psychiatry, and Psychotherapy, Hannover Medical School, Hannover, Germany

**Keywords:** panic buying, buying-shopping disorder, lockdown 2020, Germany, COVID-19

## Abstract

**Background:** During the lockdown, governmental restrictions resulted in changes to the day-to-day routines of many individuals. Some people appear to cope with stress by panic buying in an attempt to stockpile specific goods, resulting in empty supermarket shelves. Moreover, e-commerce experienced significant growth during this period. We aimed to investigate potential changes in shopping frequencies and preferred shopping type (offline/online) and their relationship with pandemic-specific anxiety and stress during the 2020 spring lockdown in Germany.

**Methods:** To address this question, we assessed self-reported changes in shopping behavior in a German sample *via* an online survey conducted during April and May 2020.

**Results:** A total of 3,122 adults were included in the analysis. Of the total sample, 35% reported no changes in their shopping behavior, 46.8% shopped less, while 18.2% shopped more during the lockdown. The groups differed with respect to sociodemographic variables, and those participants who were shopping more reported greater pandemic-related health fears and stress due to the restrictions. Moreover, they shopped online more often during the lockdown than the other two groups.

**Conclusion:** While the majority of the sample reported no changes in their shopping behavior or even shopped less during the 2020 spring lockdown, a subgroup of individuals was shopping more during this time, especially food and drugstore products. It is important to understand which factors influenced individuals to shop more so that policy makers can target this group and prevent panic buying, especially during subsequent waves of infection. It is also important to inform vulnerable persons about the risk of developing a buying–shopping disorder.

## Introduction

The rapid spread of the coronavirus disease 2019 (COVID-19) pandemic led to the widespread introduction of social distancing rules, including full lockdowns. As in the majority of countries across the world, the German government implemented a lockdown that began in all 16 partly-sovereign federal states by March 23, 2020. During this lockdown, various restrictions were imposed, such as closures of schools and non-systemically relevant facilities (e.g., restaurants, shopping malls), social and physical distancing requirements, travel bans, border closures, and cancellation of various events (e.g., cultural and sport events). These restrictions resulted in people being isolated for long periods and changes to their day-to-day routines. Since meetings with colleagues and social contacts during this period were limited, the use of Internet technology grew significantly. Video calls replaced face-to-face interactions with colleagues, family, and friends alike.

The Internet provides many opportunities for shopping where buyers can get immediate reward and emotion regulation. It provides the opportunity to buy 7 days a week/24 h a day, to shop from home and to use payment systems that can lead to inadvertent expenses (1) and, in extreme cases, to online buying–shopping disorder (2). Individuals with buying–shopping disorder are preoccupied with consumption of goods, diminished control over their spending behavior, and have the inability to normalize it even when faced with negative consequences such as debt, family conflict, and significant psychosocial impairment (3, 4). They spend money mainly for appearance-related goods or products that signal status (e.g., clothes, shoes, jewelry, makeup, art, and electronic devices) to satisfy emotional needs (1, 3–5). About one third of the patients seeking treatment for a buying–shopping disorder met the criteria for addictive online shopping (2). It is noteworthy that prevalence estimates of about 5% (6) suggest a high occurrence of buying–shopping disorder in the general population.

During the lockdown, we have encountered empty supermarket shelves. Some people seemed to cope with their stress by panic buying, attempting to stock up on toilet paper, hand soap, pasta, and other specific goods. Panic buying is explained as the “phenomenon of a sudden increase in buying of one or more essential good in excess of regular need provoked by adversity, usually a disaster or an outbreak” (7). Typical goods that are bought excessively in the context of panic buying are for instance food (e.g., rice, oil, and spices) and drugstore products (e.g., soap, toilet paper, masks, and hand sanitizers) (8). The German Federal Statistical Office reported for the period of the spring lockdown an increase in demand for soap of 337% and 221% increase in demand for toilet paper in comparison with that of the previous 6 months (9). Arafat et al. (10) analyzed English language media reports concerning aspects of panic buying and reported a sense of scarcity as being the most important cause of panic buying. Further aspects were the increased product demand, importance of the products, and anticipation of price hikes due to the pandemic. In addition, rumor, psychological factors (safety-seeking behavior, uncertainty, anxiety reduction, and taking control), social learning, lack of trust, government action, and past experience were identified as important variables that contribute to panic buying (10). The authors proposed a causal model where an adverse event or disaster (e.g., COVID-19 pandemic) causes panic buying through the abovementioned responsible factors (10). According to a recent review (11), factors that influence panic buying include the individual's perception of the health crisis and scarcity of products, fear of the unknown and negative emotions, coping behavior to relieve anxiety, and social psychological factors such as (mis)trust in government. Panic buying represents a phenomenon often seen in faces of disasters that has been investigated by different academic domains, whereas, more than three quarters (85.71%) of the research output on panic buying has occurred in the wake of the COVID-19 pandemic Arafat et al. (12).

To the best of our knowledge, it remains unclear which individuals shopped more often during the spring lockdown in Germany and how shopping behavior changed. An important question concerns potential pandemic-related changes in offline/online shopping preferences and whether the lockdown has accelerated the shift toward an increased use of digital technologies and e-commerce. While Internet use has many positive aspects (e.g., simplified access to information, increasing contact through social networks, and reducing loneliness), it can also have a negative impact on some individuals. Behavior such as gambling, use of social media and online pornography, or simply surfing the Internet can be used to reduce stress as non-problematic coping strategies but may also contribute to the development of unhealthy and potentially addictive habits (13). The same may be true for online shopping. Research indicates that specific e-commerce features such as anonymity, availability, accessibility, and affordability contribute to the development of unhealthy shopping habits or can even result in online buying–shopping disorder (1, 14–16). One may expect that many consumers switched from offline to online shopping during the lockdown due to the temporary closure of bricks-and-mortar stores. In addition, the change from offline to online shopping may have psychological aspects. Based on literature, we developed the hypothesis that in a subgroup of individuals, pandemic-specific anxiety, and stress have contributed to changes in shopping frequencies and preferences. To address this issue, we conducted an online survey investigating the relationship between self-reported changes in shopping behavior and opinions and feelings concerning the COVID-19 pandemic in a German community sample during the spring 2020 lockdown.

## Methods

This study was part of a larger online survey study conducted during the 2020 spring lockdown. Our analyses focused specifically on self-reported changes in shopping behavior during the lockdown. The survey was created using SoSci Survey Version 2.5.00-i (SoSci Survey GmbH, Munich, Germany). Aside from the assessment of changes in shopping behavior during the lockdown, participants' alcohol consumption, tobacco usage (17), media use (18), and gambling behavior as well as their eating and sport habits were investigated. The survey was promoted *via* print and social media channels as well as radio interviews and was posted from April 8 to May 11, 2020. A total of 3,122 participants (voluntary response sample) between the ages of 18 and 80 years were included in the analysis. The cover page of the survey included information about the study and its anonymous nature in accordance with the principles of the Declaration of Helsinki and the EU General Data Protection Regulation. The study protocol was approved by the ethics committee of the University of Heidelberg (registration number: 2020-552N).

### Assessment Instrument

The survey was developed in the Department of Psychiatry and Psychotherapy at the Paracelsus Medical University Nuremberg, and the Department of Addictive Behavior and Addiction Therapy at the Central Institute of Mental Health Mannheim, Germany. It included categorical assessments of several sociodemographic variables. Shopping behavior was assessed with questions concerning the preferred shopping type (preformulated answers: predominantly offline, predominantly online, both equally—online and offline) for the periods before and after the beginning of the lockdown. Participants were further asked about changes in the amount of shopping after the beginning of the lockdown (preformulated answers: a lot less, somewhat less, about the same, somewhat more, a lot more) and about concerns relating to their shopping behavior (“Have you or someone among your family and friends or a doctor been worried about your shopping behavior or suggested that you should shop less?”; preformulated answers: no concerns; yes, before the lockdown; yes, during the lockdown; yes, before and during the lockdown). Furthermore, participants were asked to answer questions regarding their opinion of the control of the pandemic in Germany (“Do you think that the corona crisis will be managed successfully in Germany?”; four-point Likert scale: 1 = certainly not, 4 = certainly yes), the importance of the restrictions (“In your opinion, are the restrictions important for the successful control of the corona virus?”; four-point Likert scale: 1 = certainly not, 4 = certainly yes), and the estimated duration of the lockdown (in weeks). Additional questions referred to pandemic-related health fears (“Are you afraid for your health or for the health of those close to you?”; 1 = not at all, 11 = yes, very much) and perceived stress due to the restrictions during lockdown (“Do you feel stressed by the restrictions?”; 1 = not at all, 11 = yes, very much).

### Statistics

Statistical analyses were conducted using the IBM SPSS statistical package, version 21.0 (IBM Corporation, Armonk, New York). Means, standard deviations, and frequencies were computed to profile the sociodemographic, shopping-specific and pandemic-related variables of the total sample and the subgroups. To assess self-reported changes in the preferred shopping type (offline vs. online vs. both equally), we performed the McNemar–Bowker test. To test for differences between the shopping groups (assembled based on the self-reported changes in shopping behavior) for categorical data, x^2^ test or—if the assumptions were not fulfilled—Fisher's exact test were performed with Cramer V as effect size. To explore significant associations between the groups and the categorical variables further, we performed *post-hoc* tests and calculated standardized residuals for the cells of the crosstabs, which quantify the standardized difference between observed and expected (from the marginal distributions) numbers. To calculate between-group differences in opinions and feelings concerning the COVID-19 pandemic, we performed multivariate analyses of variance (MANOVAs) and Bonferroni *post-hoc* analyses with $\eta_{\text{p}}^{2}$ as effect size. The significance level for all tests was set at *p* = 0.050. To counteract the problem of multiple comparisons, we used the Bonferroni–Holm method.

## Results

### Self-Reported Changes in Shopping Behavior

While 1,092 (35.0%) of the participants did not change their shopping behavior during the lockdown, 1,462 (46.8%) reported shopping less (912 somewhat less and 550 a lot less), and 568 (18.2%) reported shopping more (458 somewhat more and 110 a lot more).

### Sociodemographic Variables

Three groups were formed to compare participants who shopped more, less, and the same. The groups differed significantly concerning gender (*p* < 0.001, Cramer V = 0.08), age (*p* < 0.001, Cramer V = 0.10), years of schooling (*p* < 0.001, Cramer V = 0.07), and changes in employment status during the lockdown (*p* < 0.001, Cramer V = 0.08). Sociodemographic data are presented in Table 1. When looking at *post-hoc* tests and standardized residuals, we observed that females were found less often in the shopping same group, while males were found more often in the shopping same group and less often in the shopping less group than expected. In the shopping more group, participants between 18 and 34 years old were found more often than expected and participants over 55 years old less often. In the shopping less group, we found participants aged between 55 and 64 years old more often than expected. Participants with fewer than 11 years of schooling featured more in the shopping same group and featured less in the shopping less group, while participants with more than 13 years of schooling were found in the shopping less group more often than expected. Furthermore, participants with changes in their employment status during the lockdown were observed more often in the shopping same group.

**Table 1:** Sociodemographic description of the total sample and group differences.

	**Shopping more (*n* = 568)**	**Shopping same (*n* = 1,092)**	**Shopping less (*n* = 1,462)**		**Total sample (*N* = 3,122)**
	*n* (%)^*^; Std. Res	*n* (%)^*^; Std. Res	*n* (%)^*^; Std. Res	**Statistics**	*N* (%)^*^
**Gender**					
Female	387 (68.1)^a^; 1.2	623 (57.1)^b^; −2.9	990 (67.7)^a^; 1.7	X^2^ = 38.15^**^ df = 4 ***p*** **<** **0.001^ɫ^** Cramer V = 0.08	2,000 (64.1)
Male	178 (31.3)^a^; −1.7	467 (42.8)^b^; 3.9	469 (32.1)^a^; −2.3		1,114 (35.7)
Other	3 (0.5)^a^; 1.3	2 (0.2)^a^; −0.5	3 (0.2)^a^; −0.4		8 (0.3)
**Age**					
18–24 years old	85 (15.0)^a^; 2.1	131 (12.0)^a,b^; 0.1	156 (10.7)^b^; −1.4	X^2^ = 56.05df = 10 ***p*** **<** **0.001^ɫ^** Cramer V = 0.10	372 (11.9)
25–34 years old	188 (33.1)^a^; 2.6	302 (27.7)^b^; 0.2	363 (24.8)^b^; −1.8		853 (27.3)
35–44 years old	132 (23.2)^a^; 1.0	232 (21.2)^a^; −0.1	303 (20.7)^a^; −0.5		667 (21.4)
45–54 years old	100 (17.6)^a,b^; −1.0	192 (17.6)^a^; −1.4	314 (21.5)^b^; 1.8		606 (19.4)
55–64 years old	50 (8.8)^a^; −3.9	167 (15.3)^b^; 0.1	255 (17.4)^b^; 2.3		472 (15.1)
>65 years old	13 (2.3)^a^; −2.8	68 (6.2)^b^; 2.0	71 (4.9)^b^; 0.0		152 (4.9)
**Living arrangements**					
Alone	152 (26.8)	279 (25.6)	335 (23.1)	X^2^ = 21.07 df = 10 *p* = 0.021	766 (24.6)
With partner	178 (31.4)	410 (37.6)	487 (33.5)		1,075 (34.6)
With children	30 (5.3)	40 (3.7)	60 (4.1)		130 (4.2)
With partner and children	117 (20.6)	216 (19.8)	344 (23.7)		677 (21.8)
With parents	44 (7.8)	68 (6.2)	89 (6.1)		201 (6.5)
Other forms	46 (8.1)	77 (7.1)	137 (9.4)		260 (8.4)
**Years of schooling**					
<11 years	193 (34.2)^a^; 1.4	375 (34.7)^a^; 2.2	389 (26.8)^b^; −2.8	X^2^ = 31.24 df = 4 ***p*** **<** **0.001^ɫ^** Cramer V = 0.07	957 (30.9)
11 < x ≤ 13 years	144 (25.5)^a^; 1.0	254 (23.5)^a^; 0.0	328 (22.6)^a^; −0.7		726 (23.4)
>13 years	227 (40.2)^a^; −1.9	453 (41.9)^a^; −1.9	735 (50.6)^b^; 2.8		1,415 (45.7)
Having a systemically relevant profession^***^					
Yes	231 (41.9)	447 (42.0)	595 (41.6)	X^2^ = 0.04 df = 2 *p* = 0.978	1,273 (41.8)
No	320 (58.1)	618 (58.0)	836 (58.4)		1,774 (58.2)
**Employment status before the lockdown**					
Full-time	297 (52.4)	593 (54.4)	762 (52.2)	X^2^ = 13.52 df = 8 *p* = 0.095	1,652 (53.0)
Part-time	134 (23.6)	226 (20.7)	353 (24.2)		713 (22.9)
School/university/in training	74 (13.1)	121 (11.1)	153 (10.5)		348 (11.2)
Not working^****^	48 (8.5)	130 (11.9)	149 (10.2)		327 (10.5)
Other	14(2.5)	21 (1.9)	43 (2.9)		78 (2.5)
**Changes in employment status during the lockdown**					
Yes	264 (54.2)^a^; 2.7	426 (44.0)^b^; −0.9	578 (44.3)^b^; −0.9	X^2^ = 16.42 df = 2 ***p*** **<** **0.001^ɫ^** Cramer V = 0.08	1,268 (45.9)
No	223 (45.8)^a^; −2.5	543 (56.0)^b^; 0.8	728 (55.7)^b^; 0.8		1,494 (54.1)

* *Sums of individual items may not be equal to totals due to rounding*.

** *Fisher exact test*.

*** E.g., work in the waste management industry or a hospital or a supermarket

**** *Incl. Retired, Unemployed, Homemaker*.

ɫ *Significant after Bonferroni–Holm correction*.

*Bold values indicate significant difference*.

a,b *Values with different superscripts are significantly different (post-hoc tests)*.

### Feelings and Opinions Regarding the COVID-19 Pandemic

Results of the MANOVA with opinions and pandemic-related health fears as dependent variables suggest a significant difference between the three groups [Wilk's L = 0.97, *F*_(10,2,364)_ = 8.22, *p* < 0.001, $\eta_{\text{p}}^{2}$ = 0.02]. The ANOVA revealed a significant difference between the groups concerning participants' opinions concerning the successful control of the coronavirus [*F*_(2,2,368)_ = 6.20, *p* = 0.002, $\eta_{\text{p}}^{2}$ = 0.01], with the shopping more group (M = 2.49, SD = 0.89) reporting lower scores than the shopping less group (M = 3.09, SD = 0.79). The groups differed significantly in their opinion of the importance of the restrictions [*F*_(2,2,368)_ = 6.22, *p* = 0.002, $\eta_{\text{p}}^{2}$ = 0.01], with the shopping more group reporting lower scores (M = 3.35, SD = 0.94) than the shopping same group (M = 3.50, SD = 0.80) and the shopping less group (M = 3.51, SD = 0.80). Moreover, the groups differed regarding perceived stress due to the restrictions [*F*_(2,2,368)_ = 31.24, *p* < 0.001, $\eta_{\text{p}}^{2}$ = 0.03] and pandemic-related health fears [*F*_(2,2,368)_ = 7.78, *p* < 0.001, $\eta_{\text{p}}^{2}$ = 0.01], with the shopping more group reporting higher scores than the two other groups. The Results are presented in Table 2.

**Table 2:** Feelings and opinions regarding the COVID-19 pandemic and shopping-specific variables.

	**Shopping more (*n* = 568)**	**Shopping same (*n* = 1,092)**	**Shopping less (*n* = 1,462)**		**Total sample (*N* = 3,122)**
	**M (SD)**	**M (SD)**	**M (SD)**	**Statistics**	**M (SD)**
**Feelings and opinions regarding the COVID-19 pandemic**					
Do you agree that the corona crisis will be managed successfully in Germany? (*n* = 2,911)	2.94 (0.89)^a^	3.05 (0.81)^a,b^	3.09 (0.79)^b^	*F*_(2,2,368)_ = 6.20 ***p*** **=** **0.002** $\eta_{\text{p}}^{2}$ = 0.01	3.01 (0.83)
In your opinion, are the restrictions important for the successful control of the corona virus? (*n* = 3,022)	3.35 (0.94)^a^	3.50 (0.80)^b^	3.51 (0.80)^b^	*F*_(2,2,368)_ = 6.22 ***p*** **=** **0.002** $\eta_{\text{p}}^{2}$ = 0.01	3.42 (0.87)
In your opinion, how many weeks will the lockdown continue in its current form? (*n* = 2,545)	7.30 (8.81)	6.82 (8.37)	6.40 (7.79)	*F*_(2,2,368)_ = 2.07 *p* = 0.127	6.82 (8.38)
Are you afraid for your health or the health of those close to you? (*n* = 3,122)	6.61 (2.81)^a^	6.03 (2.70)^b^	6.07 (2.76)^b^	*F*_(2,2,368)_ = 7.78 ***p*** **<** **0.001** $\eta_{\text{p}}^{2}$ = 0.01	6.13 (2.80)
Do you feel stressed by the restrictions? (*n* = 3,096)	6.32 (3.23)^a^	4.91 (3.28)^b^	5.15 (3.13)^b^	*F*_(2,2,368)_ = 31.24 ***p*** **<** **0.001** $\eta_{\text{p}}^{2}$ = 0.03	5.42 (3.29)
**Shopping-specific variables**	***n*** **(%)**	***n*** **(%)**	***n*** **(%)**		***N*** **(%)**
Preferred shopping before lockdown					
Predominantly online	59 (10.4)^a^; 1.9	101 (9.2)^a^; 1.3	95 (6.5)^b^;−2.2	χ^2^ = 34.98 df = 4	255 (8.2)
Predominantly offline	261 (46.0)^a^; −2.2	535 (49.0)^a^; −1.7	847 (57.9)^b^; 2.8	***p*** **<** **0.001^ɫ^**	1,643 (52.6)
Both equally, online and offline	248 (43.7)^a^; 1.7	456 (41.8)^a^; 1.3	520 (35.6)^b^; 1.3	Cramer V = 0.08	1,244 (39.2)
Preferred shopping during lockdown					
Predominantly online	322 (56.7)^a^; 9.3	346 (31.7)^c^; −1.3	393 (26.9)^b^; −4.7	χ^2^ = 185.73 df = 4	1,061 (34.0)
Predominantly offline	107 (18.8)^a^; −6.9	392 (35.9)^c^; −0.2	635 (43.4)^b^; 4.5	***p*** **<** **0.001^ɫ^**	1,134 (36.3)
Both equally, online and offline	139 (24.5)^a^; −2.3	354 (32.4)^b^; 1.7	434 (29.7)^b^; 0.0	Cramer V = 0.17	927 (29.7)
Have you or someone among your family and friends or a doctor been worried about your shopping behavior or suggested that you should shop less?					
No concerns	517 (91.0)^a^; −1.2	1074 (98.4)^c^; 0.8	1409 (96.4)^b^; 0.1	χ^2^ = 81.20* df = 6	3,000 (96.1)
Yes, before the lockdown	10 (1.8)^a^; 0.1	7 (0.6)^b^; −2.7	37 (2.5)^a^; 2.3	***p*** **<** **0.001^ɫ^**	54 (1.7)
Yes, during the lockdown	20 (3.5)^a^; 6.4	1 (0.1)^b^; −2.9	8 (0.5)^b^; −1.5	Cramer V = 0.13	29 (0.9)
Yes, before and during the lockdown	21 (3.7)^a^; 5.2	10 (0.9)^b^; −1.0	8 (0.5)^b^; −2.4		39 (1.2)

ɫ *Significant after Bonferroni–Holm correction. Bold values indicate significant difference*.

a,b *Values with different superscripts are significantly different (post-hoc tests)*.

### Shopping Specific Variables

#### Worries About Shopping Behavior

Of the total sample, 96.1% reported no concerns about their shopping behavior, while 3.9% did report concerns at some point (1.7% before the beginning of the lockdown, 0.9% after the beginning of the lockdown, and 1.2% before and after the beginning of the lockdown). Participants who were shopping more, the same, or less differed significantly (x^2^ = 81.20, *p* < 0.001, Cramer V = 0.13) in their concern about their shopping behavior. When looking at *post-hoc* tests and standardized residuals, we observed that participants who reported concerns before the lockdown as well as before and during the lockdown featured more in the shopping more group and less so in the shopping less group. Participants with concerns before or during the lockdown were featured more in the shopping same group. Furthermore, participants with concerns only before the lockdown featured more in the shopping less group than expected (see Table 2).

#### Preferred Shopping Type

The groups differed significantly in their preferred shopping type before (χ^2^ = 34.98, df = 4, *p* < 0.001, Cramer V = 0.08) as well during the lockdown (χ^2^ = 185.73, df = 4, *p* < 0.001, Cramer V = 0.17). When looking at *post-hoc* tests and standardized residuals, we observed that the shopping more group featured more individuals who were buying during the lockdown predominantly online and fewer shopping online and offline equally. Moreover, participants who preferred offline shopping before the lockdown were found less often in the shopping more group. The shopping less group more often featured participants who were shopping predominantly offline before the lockdown and less often those shopping predominantly online before the lockdown. Furthermore, in the shopping less group were found fewer participants who were shopping predominantly online during the lockdown and more often those shopping predominantly offline than expected (see Table 2).

The distribution of the preferred shopping type before and after the beginning of the lockdown for the total sample differed significantly (McNemar–Bowker test x^2^ = 906.38, *p* < 0.001). The results are presented in Table 3. While the majority of the participants were shopping predominantly offline (52.6%) and only 8.2% predominantly online before the lockdown, after the beginning of the lockdown, 36.3% were shopping predominantly offline and 34.0% predominantly online.

**Table 3:** Distribution of preferred shopping before and during the lockdown (*N* = 3,122).

	**During the lockdown**			
	**Predominantly online**	**Predominantly offline**	**Both equal, online, and offline**	**Total**
**Before lockdown**				
Predominantly online	228 (7.3%)	13 (0.4%)	14 (0.4%)	255 (8.2%)
Predominantly offline	264 (8.5%)	1,029 (33.0%)	350 (11.2%)	1,643 (52.6%)
Both equal, online, and offline	569 (18.3%)	92 (2.9%)	563 (18.0%)	1,224 (39.2%)
Total	1,061 (34.0%)	1,134 (36.3%)	927 (29.7%)	3,122 (100%)

*Sums of individual items may not be equal to totals due to rounding*.

### Preferred Shopping Products of the Participants Who Shopped More

**Figure 1:**
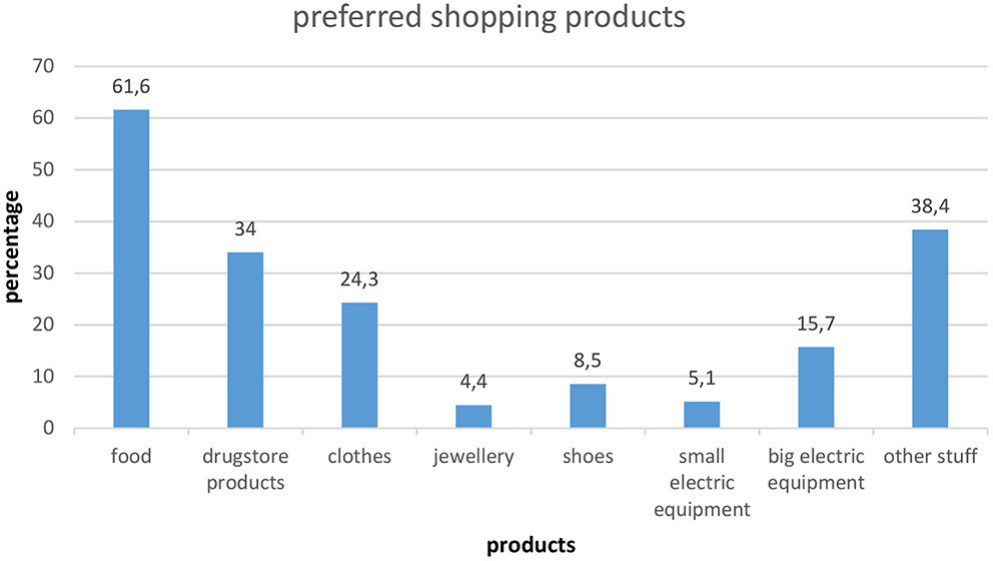
Preferred shopping products of the participants who shopped more after the beginning of the lockdown (*n* = 568). Multiple choice option.

The preferred shopping products of the shopping more group are presented in Figure 1. Participants who shopped more after the beginning of the lockdown were predominantly buying more food (61.6%). Of the 568 participants who shopped more, 38.2% (*n* = 217) reported shopping more only for food and/or drugstore products.

## Discussion

In the present study, we aimed to examine self-reported changes in shopping behavior during the 2020 spring lockdown in Germany. A majority of the participants reported no changes in their shopping behavior or shopping less during the lockdown. However, a subgroup of the total sample (18.2%) admitted shopping more, especially food and drugstore products.

A comparison of groups who shopped more, less, or the same during the lockdown showed significant differences regarding gender. Women were less likely to continue shopping the same during the lockdown. Instead, they were shopping more or less during the lockdown. In contrast, a study in Brazil (19) reported that men exhibited higher levels of panic buying than women. In our study, men were more likely to maintain their shopping behavior. It appears therefore that there were differences in shopping behavior during the pandemic between different countries, with respect to gender. Moreover, younger individuals, especially those between 18 and 34 years of age, as well as participants who reported changes in their employment status were more likely to shop more. Additionally, participants with fewer years of schooling and who may have a lower socioeconomic status (e.g., lower income and higher job insecurity) featured less in the shopping less group. Instead, they were shopping the same or even more during the lockdown. For this particular group, shopping more can lead to an additional financial burden that may, in the middle and long term, increase their concerns about their financial situation, potentially leading to greater emotional stress.

Moreover, participants who shopped more reported greater subjective stress due to the restrictions and greater pandemic-related health fears. Even though we did not assess anxiety and mood disorders with standardized questionnaires, it seems that this particular subgroup was more affected emotionally than the others. In a previous survey, researchers aimed to investigate the relationship between the perceived threat of COVID-19, personality traits, and stockpiling (toilet paper) (20). They report that the perceived threat of COVID-19 was related to toilet paper stockpiling. Emotionality (fearfulness, anxiety, dependence, and sentimentality) was associated with the perceived threat of COVID-19 and thereby indirectly affected stockpiling (20). In our sample, individuals who shopped more were less convinced that Germany would successfully manage the COVID-19 pandemic and that the restrictions taken by the government were not decisive in the control of COVID-19, when compared with individuals from the shopping same and shopping less groups. According to Yuen et al. (11), these are factors that may influence panic buying. Their perception of the health crisis, fear of unknown, negative emotions, and lower trust in the government seem to make individuals vulnerable to panic buying. Fear and anxiety are emotions that people may experience during a pandemic outbreak (21). Social and political mistrust seem to be associated with panic buying (11). Our results regarding the association between specific pandemic-related opinions/feelings and shopping more consumer goods are also in line with the model of panic buying proposed by Arafat et al. (10). The COVID-19 pandemic led to several social restrictions and individual psychological responses that may have interacted and shaped panic buying (10).

Arafat et al. (22) reported possible explanations for panic buying in society during the lockdown. They mentioned that fear of scarcity, losing control over the environment, insecurity, and social learning are factors responsible for panic buying. In our study, the most preferred products were food and drugstore goods. These products indicate that individuals were shopping more of goods that were rather necessary for their daily living. This is in line with findings from other studies, where the products that were purchased in the context of panic buying were food, drugstore, or pharmacy products (8, 23). However, in our study, only 38.2% of the shopping more group reported buying more of these products alone. The majority were also buying more of other consumer goods such as electric equipment (20.8%), clothes (24.3%), jewelry (4.4%), and shoes (8.5%). Regarding these products, it is less likely that people shopped for them through fear of empty shelves or fear of scarcity. An alternative explanation could be that they purchased such things in order to cope with feelings of losing control over the environment and insecurity. Buying large quantities of consumer goods is maladaptive (24) because it might worsen the shortage of supplies available. However, it may confer on some individuals an indirect sense of control over the situation (11), though for some others, it may cause additional worries. In our study, participants who shopped more reported more worries during the lockdown or before and during the lockdown concerning their shopping behavior than the other two groups. Concerns can arise for various reasons, e.g., implicate problematic shopping behavior, financial problems, or different opinion in the household about the amount of shopping goods that are needed or stockpiled.

As we expected, the results indicate an overall decrease in offline shopping and an increase in online shopping during the lockdown, which can be explained by the closure of many bricks and mortar shops during the lockdown (e.g., boutiques). Additionally, the shopping groups differed significantly in preferred shopping type before as well as during the lockdown. Results indicate that participants in the shopping more group were more often buying predominantly online or both equally (online and offline) before the lockdown and less often predominantly offline than the shopping less group. While the total sample showed an increase in predominantly online shopping and a decrease in predominantly offline shopping during the lockdown, we see differences between the shopping groups. While most individuals in the shopping more group were buying online, most individuals in the shopping less group were continuing to buy offline and less frequently online. On one hand, participants who shopped more might have increased or switched to online shopping in order to reduce physical contacts with other people. On the other hand, an increase in maladaptive online activities that may worsen and become addictive is currently expected by mental health professionals (25–27). While online activities and particular shopping on the Internet provide many opportunities for easy and comfortable purchasing, they can contribute to addictive shopping habits and, in some cases, even to the onset of online buying–shopping disorder (1, 13–16). Especially in people at risk from an Internet use disorder or those with a preexisting behavioral addiction (such as buying–shopping disorder), the pandemic-related restrictions may increase the severity of the problematic behavior (25). Unfortunately, we did not include standardized measures to assess specific Internet use disorders or buying–shopping disorder in our survey, which is a shortcoming.

Panic buying is a social and psychological phenomenon caused by an adverse event or disaster (10), which should be differentiated from a mental disorder (28). We expect that the majority of individuals in our study who were shopping more, especially those who were buying more food and/or drugstore goods due to homeworking or schooling, will normalize their shopping behavior after the end of the lockdown. However, we should keep in mind the possibility that some individuals became more prone to addictive shopping during the lockdown. It is worth mentioning that the buying more group reported purchasing not only food or drugstore products but also electric equipment, clothes, shoes, etc. Such non-essential goods are often excessively bought in the context of buying–shopping disorder (1, 3–5). We cannot exclude that in some participants of this group, buying more was driven by emotional and identity-related motives. Some variables that are associated with shopping more in our study are variables that are also associated with a higher risk of buying–shopping disorder. For instance, a negative emotional state (29, 30), the desire to regulate negative feelings (31), female gender (6), and younger age (6) are associated with buying–shopping disorder. Studies showed that panic buying is positively correlated with impulse buying and risk perception (19). Similarly, research indicates positive correlations between buying–shopping disorder and impulsivity (5) as well as a tendency to act rashly while in a positive or negative mood (32). Moreover, certain e-commerce features can contribute to an online buying–shopping disorder (14, 15). Suffering from a buying–shopping disorder with a predominantly online form is related to higher levels of anxiety and depression, with younger patients having a higher propensity for an online buying–shopping disorder and those who preferred online shopping being at greater risk of higher severity of buying–shopping disorder in general (2). Although for the vast majority, Internet use is adaptive and should not be pathologized, a subgroup of vulnerable individuals is at risk of developing problematic usage patterns (13).

Nevertheless, consumers are likely to learn or develop new shopping routines due to a crisis such as the COVID-19 pandemic (33). Lockdown, isolation, loss of employment, financial insecurity, and stress can contribute to a fertile terrain in which behavioral addictions flourish (34). Also, the unavailability of many bricks and mortar shops during the lockdown and the many opportunities afforded by e-commerce can contribute to higher risks of developing a buying–shopping disorder that can lead to severe consequences for some individuals and should therefore not be underestimated. Availability of accurate information to the public can reduce both panic buying and risk for shopping addiction. The (social) media have a critical role in influencing a crisis. Pictures of empty shelves can increase fear of scarcity that may encourage panic buying. A group of multidisciplinary and multinational experts in the problematic usage of the Internet have made practical recommendations that may help to reduce the risks of increased, maladaptive online activities (13). Promoting shopping as a coping strategy or commercial messages such as “maintaining distance is easier online” can contribute to some individuals suffering harm in times of crisis.

## Limitations

Our results should be interpreted in the context of certain limitations. Although the results presented are derived from a large number of participants from the general population, it must be kept in mind that the sample in our survey is a voluntary response sample with a relatively high proportion of young, well-educated females, and the recruiting methods exclude the “offline” population that does not use the Internet. Because of the sampling method, no information about non-responders is available, which may affect the generalizability of our findings (35). Furthermore, the cross-sectional design of our study prevents any causal interpretation, and reported effect sizes are small to moderate. We did not assess information about the motivation for shopping more during the lockdown and have not used standardized questionnaires. Future longitudinal studies should address both risks as well as protection factors for panic and addictive buying during lockdown situations, investigate the long-term effects of panic buying, and investigate the phenomenological characteristics, especially of participants who are at risk of developing an (online) buying–shopping disorder.

## Conclusion

While supermarket shelves were empty during the lockdown, we would expect this to be the result of many people stockpiling. However, our results indicate that this is a consequence of the shopping behavior of a subgroup of people. Moreover, this subgroup was shopping more food and drugstore products and reported greater subjective stress due to the restrictions and greater pandemic-related fears. Furthermore, results indicate an overall decrease in offline shopping and an increase in online shopping during the lockdown. It is important to understand which factors influence those people to shop more so that policy makers can target this group and prevent panic buying—especially in the case of subsequent waves of infection—and also to inform them about the risks of developing a buying–shopping disorder.

## Data Availability Statement

The raw data supporting the conclusions of this article will be made available by the authors, without undue reservation.

## Ethics Statement

The study protocol was approved by the ethics committee of the University of Heidelberg (registration number: 2020-552N). The patients/participants provided their consent to participate in this study.

## Author Contributions

EG analyzed data and prepared the first draft. All authors designed the survey, provided comments, and revised the manuscript.

## Conflict of Interest

The authors declare that the research was conducted in the absence of any commercial or financial relationships that could be construed as a potential conflict of interest.
